# Integrated effects of compost and nano foliar spray on the growth and fruit quality of sweet peppers under greenhouse conditions

**DOI:** 10.3389/fpls.2026.1743784

**Published:** 2026-01-27

**Authors:** Hassan A. A. Sayed, Khaled A. M. Ali, Mahmoud A. Abdelhamid, Ali S. A. Nofal, Saleh A. S. Hamad, Qinghui Lai, Mohamed Ahmed Moustafa, Gomaa G. Abd El-wahhab

**Affiliations:** 1 School of Energy and Environment Science, Yunnan Provincial Rural Energy Engineering Key Laboratory, Yunnan Normal University, Kunming, China; 2 Department of Agricultural Machinery and Power Engineering, Faculty of Agricultural Engineering, Al-Azhar University, Cairo, Egypt; 3 College of Engineering, South China Agricultural University, Guangzhou, China; 4 Department of Agricultural Engineering, Ain Shams University, Faculty of Agriculture, Cairo, Egypt; 5 Department of Horticulture, Faculty of Agriculture, Al-Azhar University, Cairo, Egypt; 6 Faculty of Agricultural Engineering, Al-Azhar University, Cairo, Egypt; 7 College of Engineering, Nanjing Agricultural University, Nanjing, China; 8 Department of Agricultural Construction Engineering and Environmental Control, Faculty of Agricultural Engineering, Al-Azhar University, Cairo, Egypt

**Keywords:** compost, nano-fertilizer, nutrient assimilation, plant physiology, sustainable greenhouse cultivation

## Abstract

**Introduction:**

Enhancing vegetable growth and fruit quality in greenhouse production systems through sustainable nutrient management is a key challenge in modern horticulture. Therefore, this study evaluated the effects of mechanically produced compost and nano-foliar spray application on the growth and fruit quality of sweet pepper (*Capsicum annuum L*.) grown in a greenhouse under sand and clay soil conditions.

**Methods:**

The experiment was conducted in a controlled plastic greenhouse located at Al-Azhar University, Cairo, Egypt (30° 2′ 44′′ N, 31° 15′ 44′′ E), over the 2022–2023 growing season. It was conducted using a factorial design consisting of compost at five volumetric rates (0%, 10%, 15%, 20%, and 25%), two soil types (sand and clay), and nano foliar spray at three concentrations (0, 1, and 2 cm^3^ L^–1^), with three replicates per treatment. Plant growth characteristics and fruit quality parameters were measured. The data were analyzed using three-way analysis of variance, and treatment means were compared using Duncan’s multiple range test (p ≤ 0.05).

**Results:**

The results indicated that the level of compost, soil type, and foliar nano-spray significantly affect pepper growth and fruit quality. The treatment (20% compost in sandy soil + 2 cm^3^ L^–1^ of nano-fertilizer) showed better performance in most vegetative growth characteristics and fruit quality traits compared to the other treatments, with a yield of 72.4 tons/ha. Furthermore, Multivariate analyses, including Principal Component Analysis (PCA) and correlation analysis, highlighted strong associations between yield and physiological traits related to photosynthetic capacity and antioxidant status.

**Discussion:**

The proposed approach highlights the integration of organic amendments and nano-based nutrient management as an effective strategy to increase productivity and produce more sustainable vegetables.

## 1 Introduction

Sweet pepper (*Capsicum annuum L.*) is a high-value horticultural crop widely cultivated in greenhouse systems due to its nutritional richness in vitamin C, carotenoids, and minerals, as well as its economic importance in global vegetable markets (Choudhary et al., 2024; Moustafa et al., 2024; Sobczak-Samburska et al., 2025). However, intensive greenhouse cultivation often depends on continuous fertilizer inputs, which can lead to nutrient imbalances, low use efficiency, and environmental degradation through leaching and soil salinization. Developing sustainable nutrient management strategies that optimize both plant nutrition and soil health has therefore become a major goal in modern horticultural systems. Organic amendments such as compost play a central role in improving soil fertility, microbial activity, and the overall nutrient status of greenhouse crops (Akande et al., 2023; EL-Mogy et al., 2024; Abd El-Lateef et al., 2025). Compost enhances the soil’s physical structure, increases cation exchange capacity (CEC), and supports beneficial microbial communities that promote nutrient mineralization and root nutrient uptake (Arancon et al., 2003; Jamir et al., 2017; Azim et al., 2018; Ou-Zine et al., 2021; Amirahmadi et al., 2024). Mechanically produced compost, developed using self-propelled or aerated turning systems, offers superior homogeneity and faster stabilization compared with manually produced compost (Rokach and Maimon, 2006; Reitbauer et al., 2020; Schedler et al., 2020; Cichocki et al., 2024). The engineered compost turning machine (CTM) described by Sayed et al. (2022) improves aeration and microbial activity, resulting in compost with an optimal C/N ratio (≈15:1) and high organic matter content, suitable for sustainable horticultural use. This mechanization of composting also supports circular agriculture principles by recycling agricultural residues into high-quality organic fertilizers.

In parallel, nano-fertilizers have emerged as a promising innovation in plant nutrition management (Ali et al., 2025; Sheta et al., 2025). Due to their ultra-small particle size, large surface area, and enhanced reactivity, nano-nutrients can penetrate plant tissues efficiently through stomatal openings, improving nutrient absorption and translocation (Mastronardi et al., 2015; Neme et al., 2021; Beig et al., 2022; Soni et al., 2024). Compared to conventional fertilizers, nano-fertilizers enable controlled nutrient release, reduce losses, and enhance photosynthetic and physiological responses (Davari et al., 2017; García-Gómez et al., 2020; Avila-Quezada et al., 2022; Channab et al., 2024). Elements such as nano-calcium and nano-boron play critical physiological roles: calcium stabilizes cell walls and membranes, while boron regulates pollen viability, carbohydrate translocation, and fruit set (Davis et al., 2003; Hepler and Winship, 2010; Esringü et al., 2011; Youssef et al., 2017). However, while many studies have explored compost or nano-fertilizer effects separately, limited research has examined their integrated application and the synergistic mechanisms that may arise from combining root-supplied organic nutrients with foliar nano-scale delivery systems.

The integration of organic compost and nano-fertilizer represents a potentially powerful approach to enhancing nutrient assimilation and photosynthetic metabolism in crops. Compost improves the root-zone environment by enhancing moisture retention and microbial-driven nutrient release, while nano-fertilizers ensure rapid foliar nutrient delivery and metabolic activation. This combination may lead to balanced nutrient availability, optimized ion homeostasis, and improved metabolic performance under variable soil textures. Furthermore, sandy soils in particular benefit from compost additions due to their low native fertility and poor water-holding capacity, suggesting that compost–nano integration could compensate for such limitations through complementary nutrient pathways (Easwaran et al., 2024; Faizan et al., 2024; Hameed and Ashfaq, 2024; Hoque et al., 2024; Mohd Noor and Elgharbawy, 2024; Rasool et al., 2024; Meng et al., 2025; Shaheen and Abdelwahab, 2025).

This study aims to evaluate the combined effects of machine-generated compost and nano-fertilizer on the physiological, biochemical, and agronomic traits of greenhouse-grown sweet pepper and elucidate how these treatments influence nutrient assimilation and photosynthetic metabolism across contrasting soil textures. This research contributes to the development of sustainable, physiology-based nutrient management frameworks aligned with the United Nations Sustainable Development Goals (SDG 13 and 15).

## 2 Materials and methods

### 2.1 Experimental site and plant material

The experiment was conducted in a controlled plastic greenhouse located at Al-Azhar University, Cairo, Egypt (30° 2′ 44″ N, 31° 15′ 44″ E), over the 2022–2023 growing season. The site is situated in a hot desert climate (BWh, Köppen classification), characterized by long, hot summers and mild winters. Average annual temperatures range from 13.5 °C in January to 35.2 °C in July, with relative humidity fluctuating between 50% and 60%, and low annual precipitation (20–25 mm), mostly concentrated in the winter months. Sweet pepper (*Capsicum annuum L.*), hybrid F1 cultivar 702, was selected for this study due to its suitability for greenhouse cultivation. Seeds were sown in 1:1 peat moss–vermiculite trays and grown in a nursery for 40 days before transplanting on November 5, 2022. Each pot contained a single seedling. In winter, greenhouses were covered with 120-micron white polyethylene plastic, while in summer, shading nets were used to moderate temperature and light.

### 2.2 Experimental process and workflow

**Figure 1:**
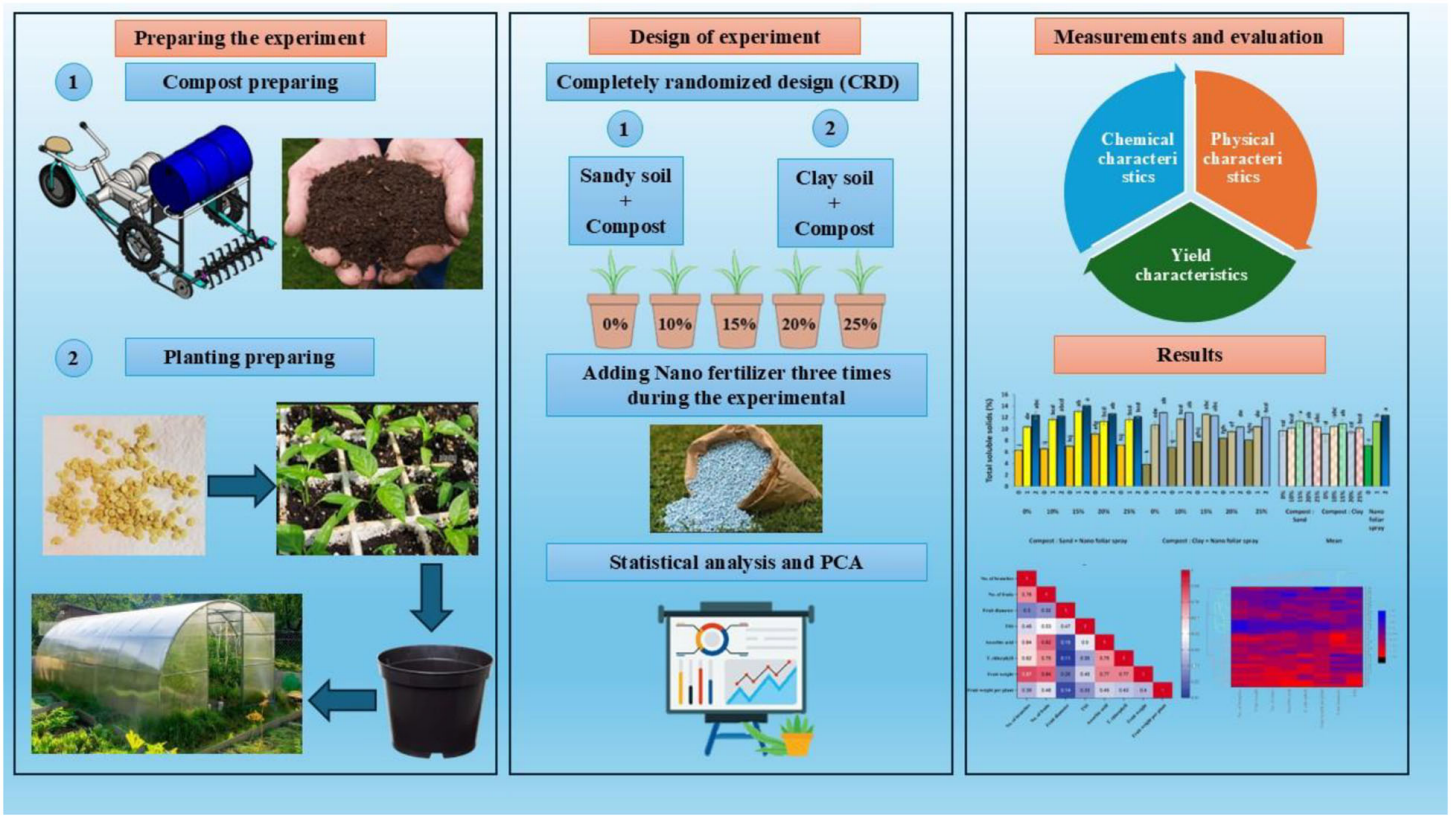
The outline of the experiment.

The experimental workflow was designed to evaluate the integrated effects of compost and nano-fertilizer application on sweet pepper growth under greenhouse conditions. The process started with mechanical compost preparation and soil amendment, followed by transplanting uniform seedlings into pots and applying the experimental treatments according to a factorial arrangement. Plants were subsequently maintained under standard greenhouse management, during which physiological, biochemical, and yield-related traits were monitored. At harvest, data were collected and subjected to statistical and multivariate analyses to assess treatment effects and relationships among measured traits. The outline of the experimental work is shown in **Figure 1**.

#### 2.2.1 Compost production and characterization

**Figure 2:**
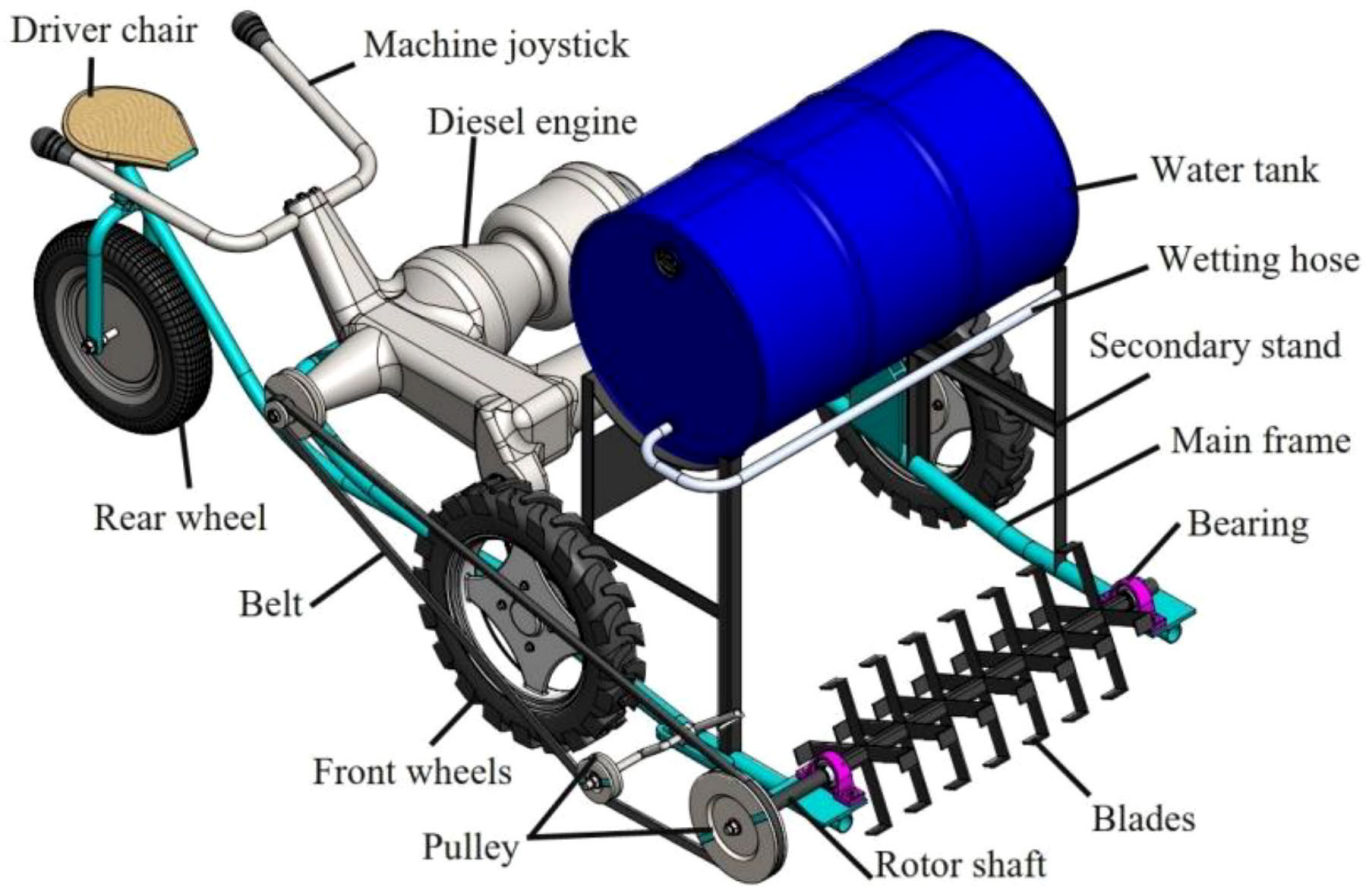
The CTM component, according to Sayed et al. (2022).

To produce high-quality compost with improved efficiency and reduced labor requirements, a locally developed CTM was utilized in this study (**Figure 2**). The CTM was adapted from a self-propelled harvesting chassis and optimized specifically for smallholder use, offering low-cost and scalable composting solutions. The CTM was developed at the Al-Hosayneya Center in Sharkeya Governorate, Egypt. Structurally, the CTM consists of a cylindrical steel mainframe mounted on three wheels and powered by a 15 hp (11.5 kW) diesel engine. The central component is a rotating shaft (160 cm in length, 4 cm in diameter) fitted with seven blade sets (four blades per set), with L-shaped blades, designed to optimize mixing and aeration. The turning rotor diameter is 40 cm, with a maximum turning width of 120 cm and a height of 100 cm. The shaft receives torque via a belt-and-pulley mechanism and is supported by ball bearings to reduce friction and ensure rotational stability. The CTM was developed by Sayed et al. (2022). The CTM features a rotating shaft equipped with adjustable mixing fingers and an integrated aeration system, enabling uniform oxygen distribution and temperature control during the composting process. The system was specifically optimized to enhance microbial activity and decomposition efficiency of agricultural residues, resulting in high-quality compost with a carbon-to-nitrogen (C:N) ratio of 15.3:1, 35% total organic matter, and a water-holding capacity of 4.1 g water/g dry compost.

Compost quality parameters, including pH, electrical conductivity (EC), total nitrogen, organic carbon, and moisture content, were analyzed according to standard procedures (Azim et al., 2018). **Table 1** compares the physicochemical properties of compost produced via the CTM method with those produced manually, confirming the mechanical system’s superior efficiency and consistency.

**Table 1:** Compost’s chemical and physical properties for machine and manual production.

Properties	Ideal range	Compost of the machine	Manual compost
C:N ratio	14:1 - 18.5:1	15.3:1	19:1
Total organic matter, %	28.6 - 41.2	35	29.4
Total organic carbon, %	16.6 - 23.89	17.6	19.2
Total nitrogen, %	0.95 - 1.68	1.15	1
Moisture content, %	23.5 - 32.1	25.5	28.7
Water holding capacity, g water/g dry	3.5 - 4.4	4.1	3.8
pH	6.3 - 7.8	7.8	7.5
EC, dS/m	2.6 - 4.1	3.62	3.44

#### 2.2.2 Experimental design and treatments

A completely randomized design (CRD) in a factorial arrangement was implemented with three replications (three pots) per treatment, resulting in three experimental units per treatment. Each pot (25 cm height × 20 cm diameter) was filled with soil-compost mixtures at specified volumetric ratios. Each pot containing one plant was considered the experimental unit. Therefore, the experiment followed a 3-factor factorial structure: two soil types (sandy and clay), five compost rates (0%, 10%, 15%, 20%, and 25% by volume), and three nano-fertilizer concentrations (0, 1, and 2 cm^3^ L^-1^). The experiment involved a total of 30 treatment combinations, as detailed in **Table 2**. Each treatment replicate was three pots, and all pots were arranged randomly in the greenhouse to minimize environmental variability. To mitigate the impact of ecological microvariations within the greenhouse, all pots were arranged in a completely randomized configuration at the start of the experiment. This method mitigated potential positioning influences and confirmed that any detected variations were due to treatment parameters rather than microenvironmental fluctuations.

**Table 2:** Treatments used during the experiment.

Treatment	Soil type	Compost (%)	Nano foliar spray (cm^3^ L^-1^)	Treatment	Soil type	Compost (%)	Nano foliar spray (cm^3^ L^-1^)
T1	Sand	0	0	T16	Clay	0	1
T2	Sand	10	0	T17	Clay	10	1
T3	Sand	15	0	T18	Clay	15	1
T4	Sand	20	0	T19	Clay	20	1
T5	Sand	25	0	T20	Clay	25	1
T6	Clay	0	0	T21	Sand	0	2
T7	Clay	10	0	T22	Sand	10	2
T8	Clay	15	0	T23	Sand	15	2
T9	Clay	20	0	T24	Sand	20	2
T10	Clay	25	0	T25	Sand	25	2
T11	Sand	0	1	T26	Clay	0	2
T12	Sand	10	1	T27	Clay	10	2
T13	Sand	15	1	T28	Clay	15	2
T14	Sand	20	1	T29	Clay	20	2
T15	Sand	25	1	T30	Clay	25	2

The nano-fertilizer used (“Nano Hiber feed”) was obtained from Nano FAB Technology, 6th October City, Cairo, Egypt. Its composition included 10% Nano-Nitrogen (N), 10% Nano-Calcium (Ca), 2% Nano-Magnesium (Mg), and 0.05% Nano-Boron (B). The nano-fertilizer used in this study was a commercially available product obtained directly from the manufacturer and applied in its ready-to-use form with an average particle size of 50–80 nm and verified stability under standard storage conditions. Foliar applications were performed three times per season:

- 21 days after transplanting.
- At full flowering.
- Three weeks post-flowering.

Foliar spraying was performed using a handheld sprayer until runoff, applying concentrations of 1 and 2 cm^3^ L^-1^ as per the treatment design, while control plants received only tap water.

#### 2.2.3 Data collection and measurements

All physiological, biochemical, and yield measurements were conducted on all plants (three plants) per treatment, corresponding to the three replicated pots. The growth and yield measurements were:

- Number of fruits per plant.
- Average fruit diameter (cm).
- Average fruit weight (g).
- Total fruit yield (ton/ha).
- Calculated total yield per hectare.

While the fruit quality measurements included:

- Ascorbic acid content (mg/100 g FW) was determined using 2,6-dichlorophenolindophenol titration (Dinesh et al., 2015).
- Total soluble solids (TSS) were measured in freshly extracted juice using a digital refractometer (AOAC, 1999).
- Total chlorophyll content was assessed non-destructively using a SPAD-502 Plus chlorophyll meter (Konica Minolta), indicating relative greenness and nitrogen status (Pôrto et al., 2014).

#### 2.2.4 Statistical analysis

Data were analyzed using a three-way factorial ANOVA (soil type × compost rate × nano-fertilizer concentration) in a CRD using CoStat v6.4 (CoHort Software, USA). Main effects and all interaction effects among the three factors were tested. When significant differences were detected (p < 0.05), means were separated using Duncan’s Multiple Range Test. Principal Component Analysis (PCA) and hierarchical clustering (heatmaps) were performed using R software (v4.3.0).

## 3 Results

The integration of compost and nano-fertilizer applications demonstrated significant and consistent effects across various growth, yield, and quality parameters of sweet pepper cultivated under greenhouse conditions. The results reflect the effectiveness of combining a high-quality organic amendment, produced by a novel CTM, with nano-scale nutrient delivery technologies. This dual-input strategy represents a promising sustainable approach for enhancing productivity in protected horticultural systems.

### 3.1 Effects on nutrient assimilation and photosynthetic performance

**Figure 3:**
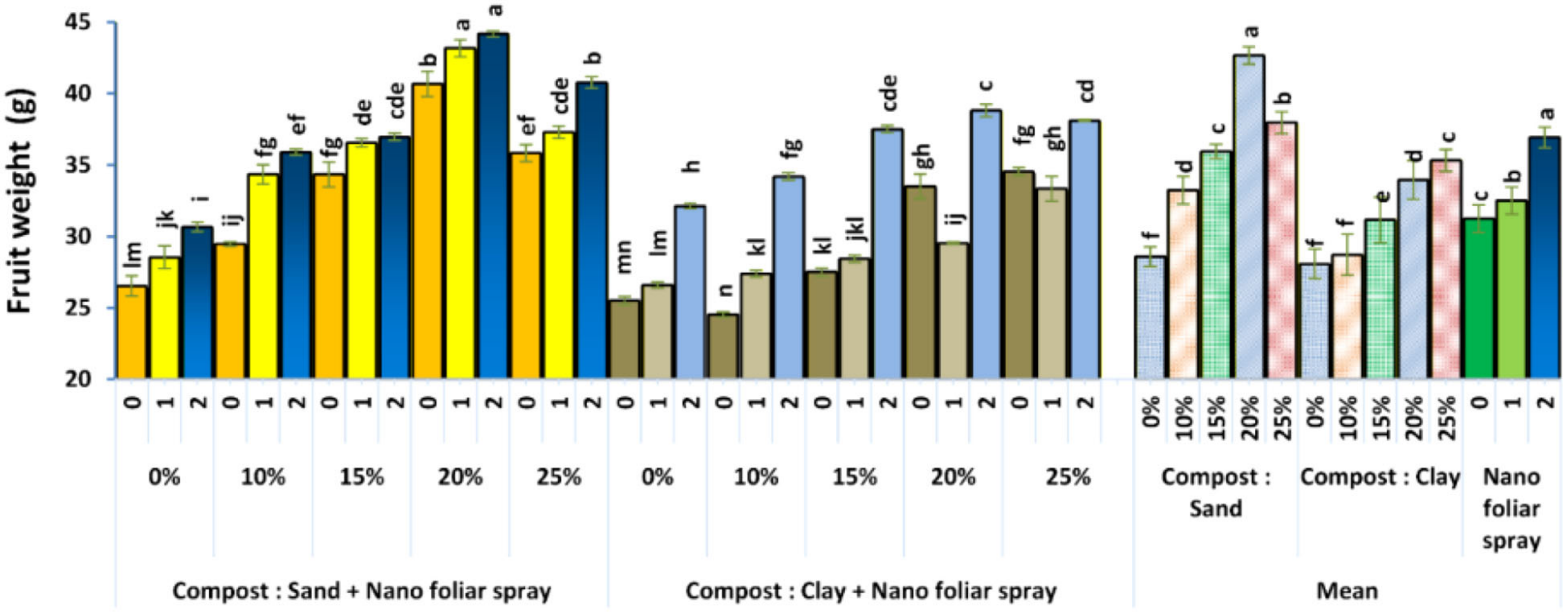
Effect of soil culture and compost combined with foliar application of nano fertilizer on fruit weight (g). Different letters indicate statistically significant differences at p < 0.05 according to Duncan’s Multiple Range Test (n = 3 replicates per treatment).

Sweet pepper vegetative growth and average fruit weight (**Figure 3**) improved significantly with compost amendment, especially when combined with 2 cm^3^ L^-1^ nano-fertilizer. The highest fruit mass occurred in sandy soils with 20–25% compost. These gains are attributed to enhanced soil moisture retention, greater nutrient availability, and foliar nano-calcium and nano-nitrogen supply, which may stimulate cell division and expansion. The combined root and foliar nutrient delivery created an optimal nutrient environment, consistent with EL-Mogy et al. (2024), who reported similar benefits from integrating organic and nano-based amendments.

**Figure 4:**
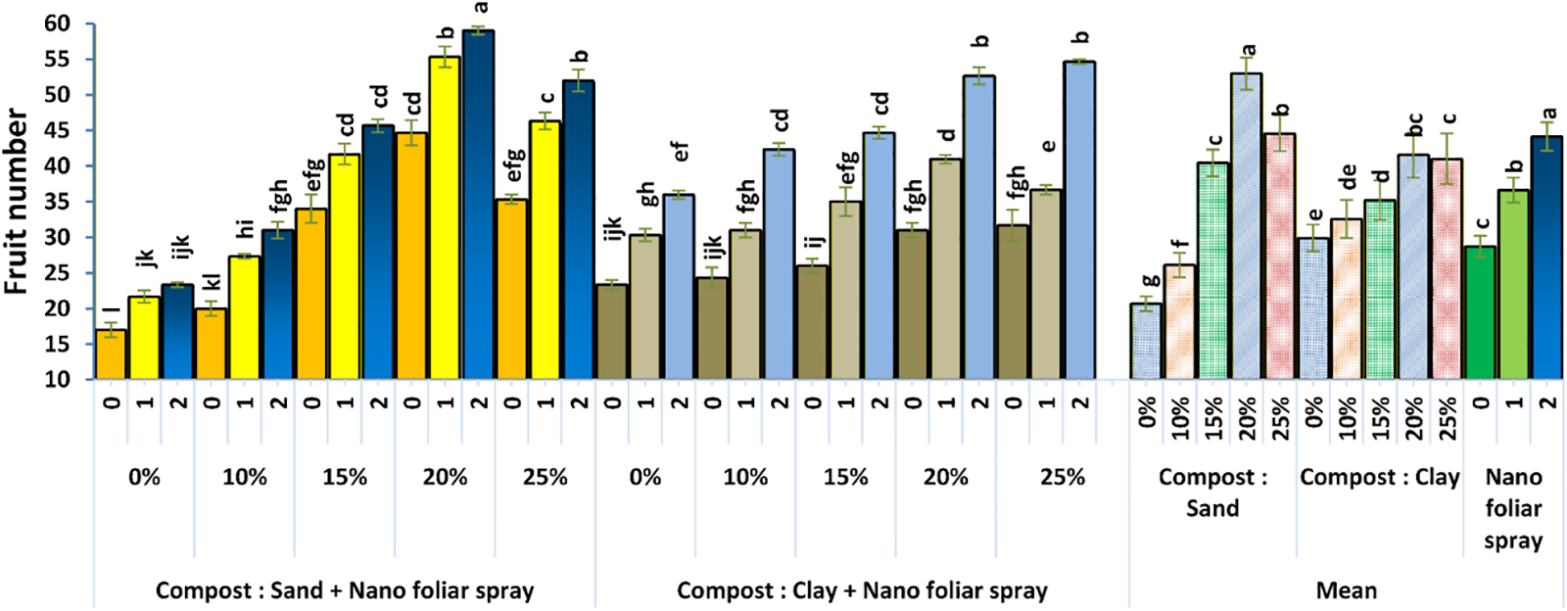
Effect of soil culture and compost combined with foliar application of nano fertilizer on plant fruit number. Different letters indicate statistically significant differences at p < 0.05 according to Duncan’s Multiple Range Test (n = 3 replicates per treatment).

The number of fruits per plant (**Figure 4**) was highest in sandy soil with 20% compost plus 2 cm^3^ L^-1^ nano-fertilizer. This likely reflects improved flowering, pollination, and fruit set due to enhanced nutrient status. Boron in the nano-fertilizer may have supported pollen viability and hormone regulation, while compost-stimulated soil microbiota could have improved nutrient mobilization. These results align with those of Esringü et al. (2011), who reported increased fruit set and yield in strawberries and tomatoes following boron application.

**Figure 5:**
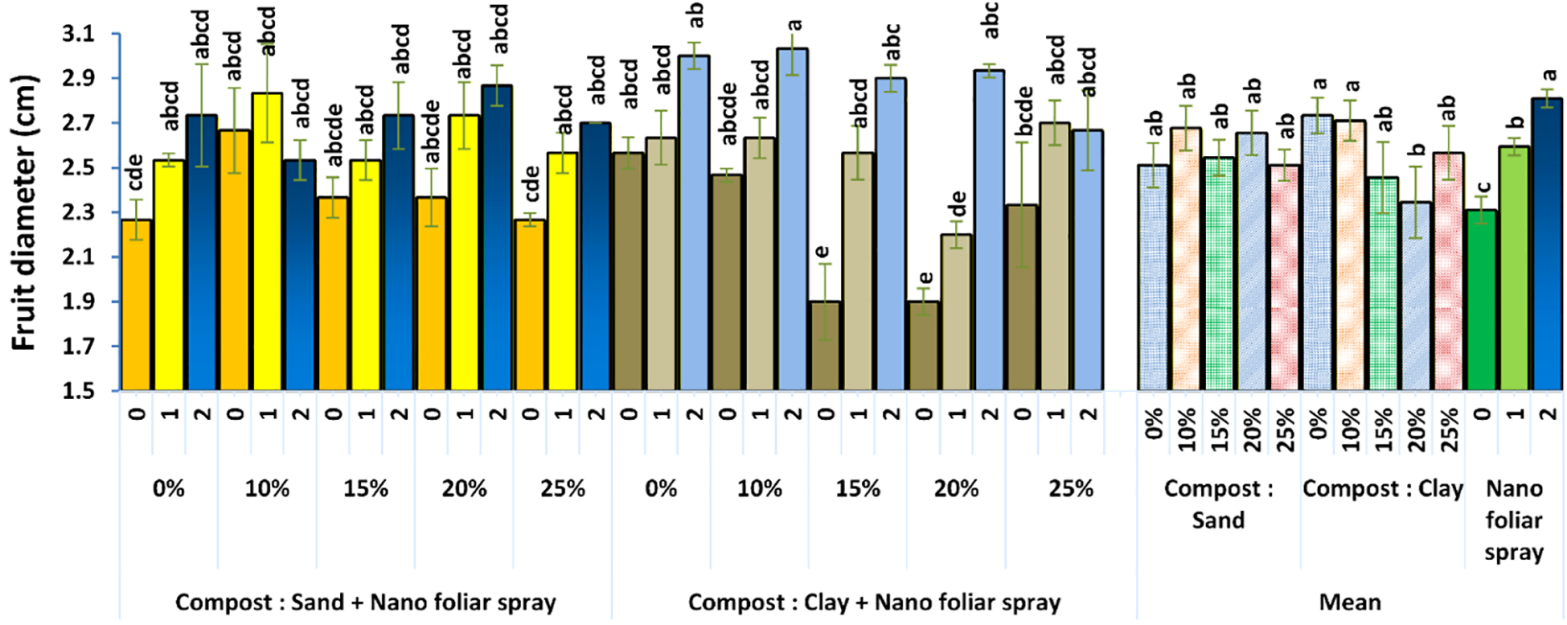
Effect of soil culture and compost combined with foliar application of nano fertilizer on plant fruit diameter (cm). Different letters indicate statistically significant differences at p < 0.05 according to Duncan’s Multiple Range Test (n = 3 replicates per treatment).

In contrast, fruit diameter (**Figure 5**) showed no statistically significant differences among compost levels alone, but slight improvements were observed when nano-fertilizer was applied. This suggests that while compost improved general growth conditions, the foliar-applied nutrients played a more critical role in stimulating cell elongation and fruit expansion. The modest increase could be associated with calcium’s role in maintaining cell wall integrity and osmotic regulation (Hepler and Winship, 2010). Though the diameter differences were not dramatic, even marginal improvements in marketable fruit size can have significant economic implications.

**Figure 6:**
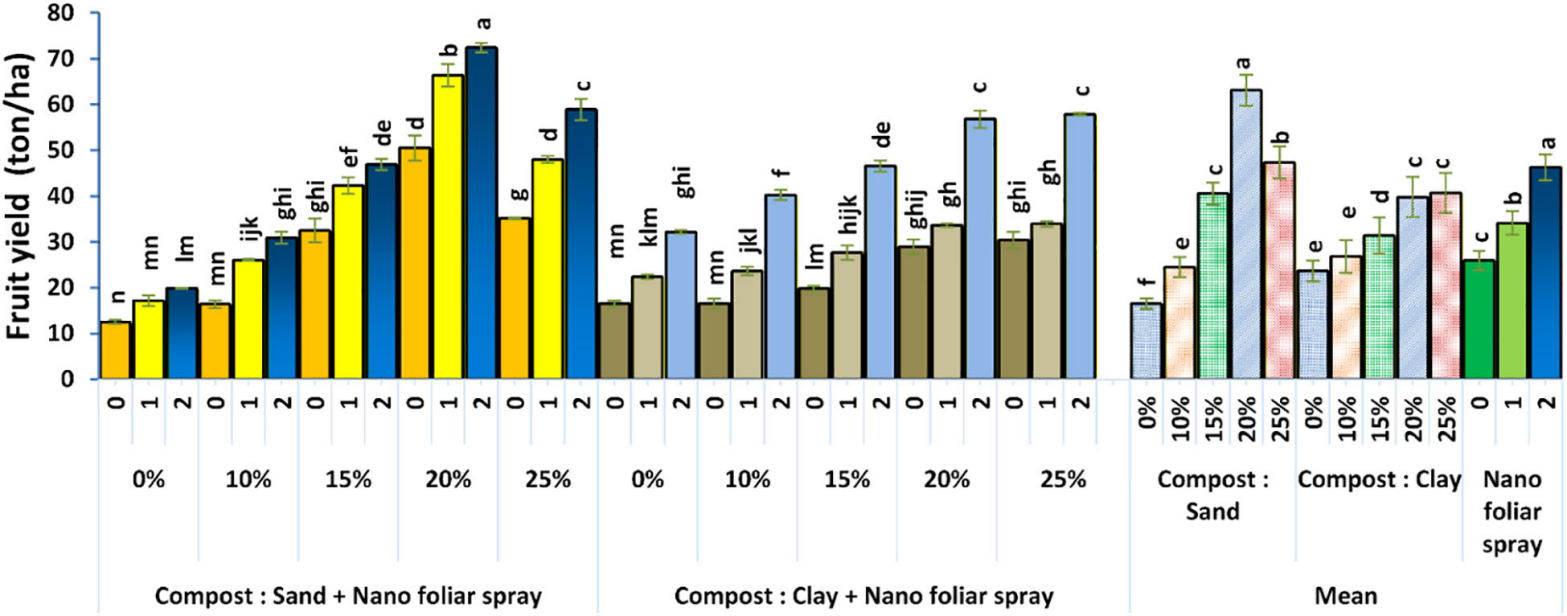
Effect of soil culture and compost combined with foliar application of nano fertilizer on total yield (ton/ha). Different letters indicate statistically significant differences at p < 0.05 according to Duncan’s Multiple Range Test (n = 3 replicates per treatment).

The total yield per plant, as depicted in **Figure 6**, reflects the cumulative effect of improved vegetative growth, fruit set, and fruit size. The highest yield was achieved with the application of 20% compost in sandy soil and 2 cm^3^ L^-1^ nano-fertilizer. This outcome confirms the agronomic effectiveness of the combined strategy, particularly in soils with limited resources. The compost’s capacity to improve soil water retention and root environment, along with the nano-fertilizer’s efficiency in nutrient delivery, synergistically enhanced the crop’s productivity. This finding aligns with that of EL-Mogy et al. (2024), who found that integrating compost and biochar enhanced yield components in greenhouse peppers.

### 3.2 Modulation of chlorophyll, antioxidant activity, and metabolic quality

**Figure 7:**
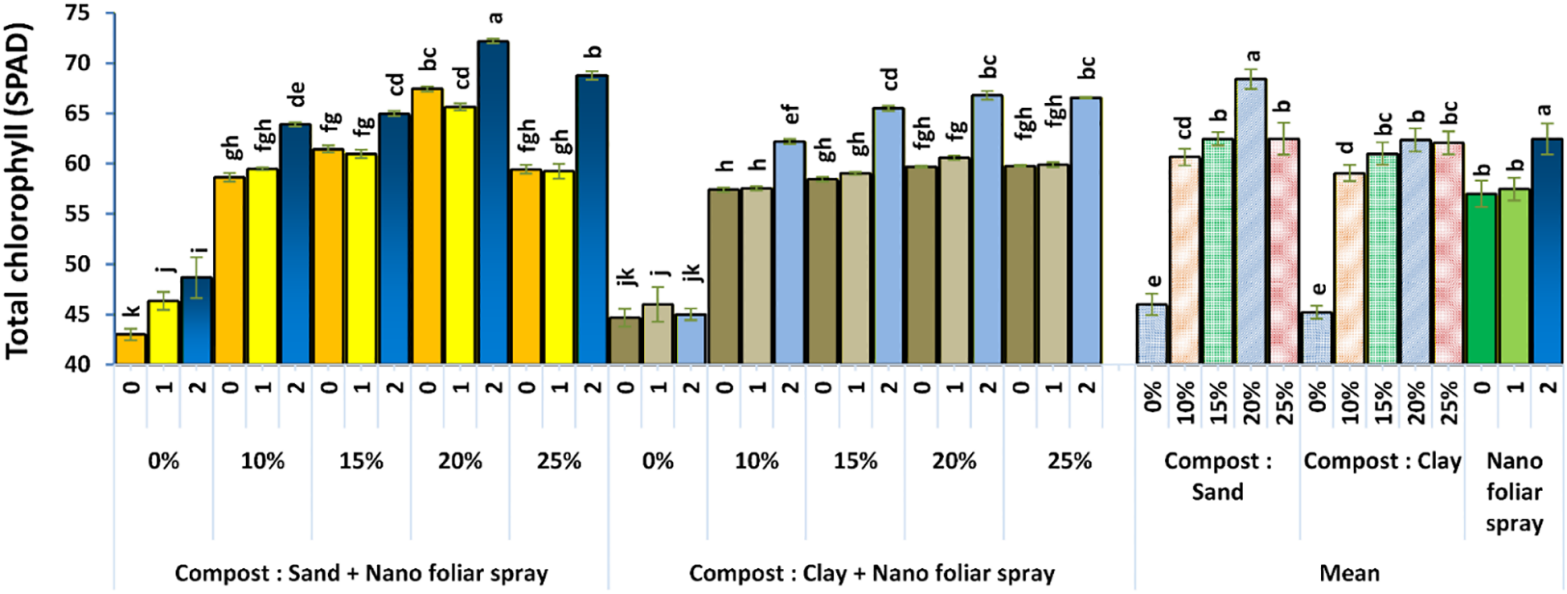
Effect of soil culture and compost combined with foliar application of nano fertilizer on total chlorophyll (SPAD). Different letters indicate statistically significant differences at p < 0.05 according to Duncan’s Multiple Range Test (n = 3 replicates per treatment).

Regarding biochemical traits, total chlorophyll content (SPAD index, **Figure 7**) increased significantly with the addition of compost and nano-fertilizer, especially at a concentration of 2 cm^3^ L^-1^. The highest SPAD values were observed in sandy soils with 20% compost, indicating enhanced photosynthetic activity and nitrogen assimilation through nano-nitrogen supply, as well as improved soil fertility. These findings align with those of Pôrto et al. (2014), who validated SPAD as an indicator of nitrogen status in vegetables, and with Amirahmadi et al. (2024), who reported yield gains driven by compost and reduced ecosystem impacts in wheat systems.

**Figure 8:**
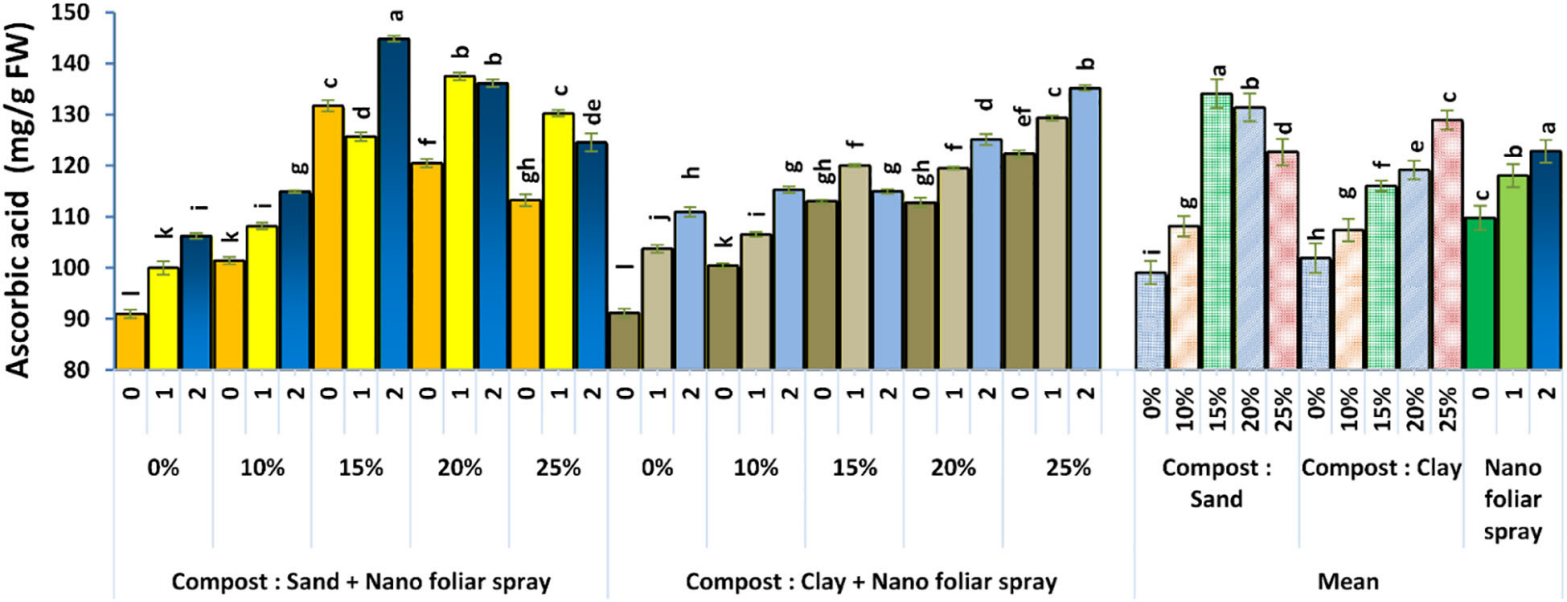
Effect of soil culture and compost combined with foliar application of nano fertilizer on fruit ascorbic acid content. Different letters indicate statistically significant differences at p < 0.05 according to Duncan’s Multiple Range Test (n = 3 replicates per treatment).

Fruit ascorbic acid content, shown in **Figure 8**, was significantly improved by the integrated application of compost and nano-fertilizer. The highest ascorbic acid levels were recorded in plants grown in sandy soil with 15% compost and 2 cm^3^ L^-1^ nano-fertilizer, followed closely by those grown in clay soil with 25% compost under the same foliar treatment. These increases can be attributed to enhanced metabolic activity and stress resilience, as compost improves antioxidant enzyme activity and nano-fertilizers boost micronutrient uptake. Ascorbic acid is a key quality parameter that enhances the nutritional and commercial value of sweet pepper. This result aligns with Dinesh et al. (2015) and Youssef et al. (2017), who highlighted the role of micronutrients in stimulating the biosynthesis of secondary metabolites in vegetables.

**Figure 9:**
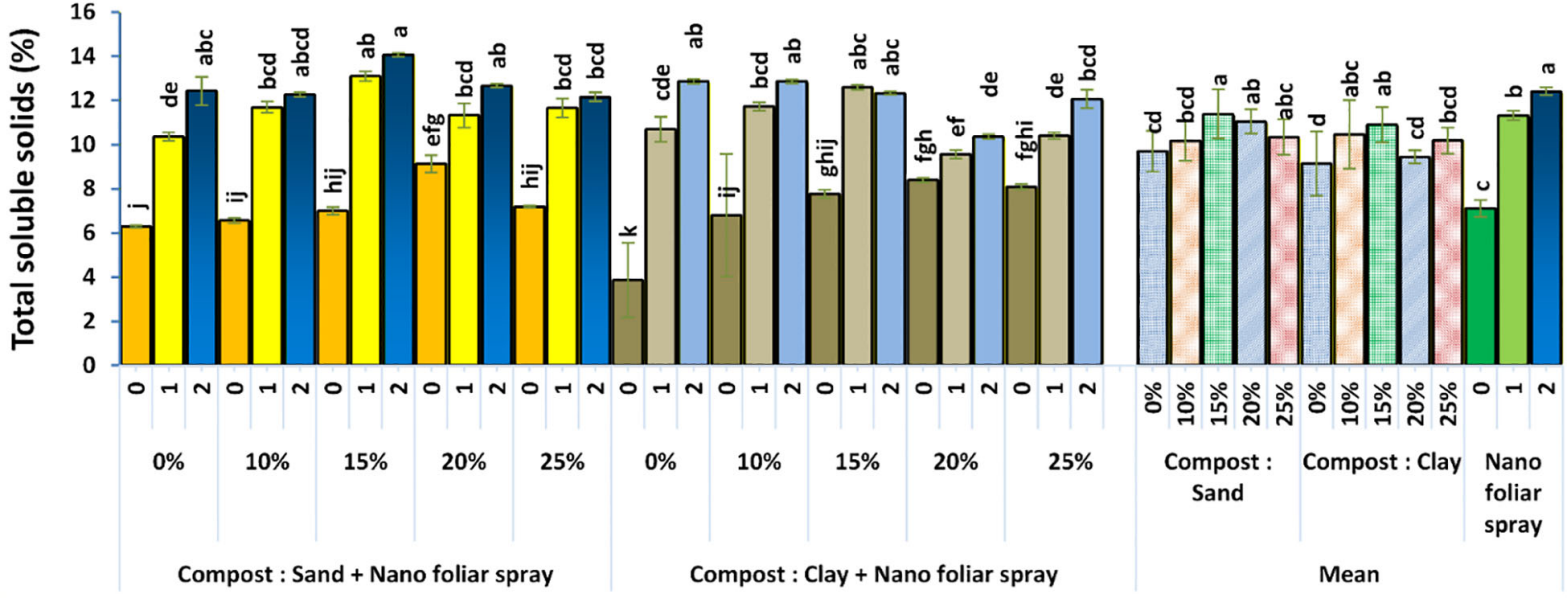
Effect of soil culture and compost combined with foliar application of nano fertilizer on fruit total soluble solids. Different letters indicate statistically significant differences at p < 0.05 according to Duncan’s Multiple Range Test (n = 3 replicates per treatment).

Similarly, **Figure 9** shows that the total soluble solids (TSS) content was significantly higher in fruits from plants grown with 15% compost and treated with 2 cm^3^ L^-1^ nano-fertilizer. This effect was particularly pronounced in sandy soil. The elevated TSS levels suggest improved carbohydrate accumulation, likely driven by enhanced photosynthetic efficiency and nutrient translocation under optimal nutritional conditions. These findings suggest that integrated nutrient management not only enhances yield but also improves taste and shelf life, two key attributes for market competitiveness.

### 3.3 Multivariate relationships and overall treatment performance

**Figure 10:**
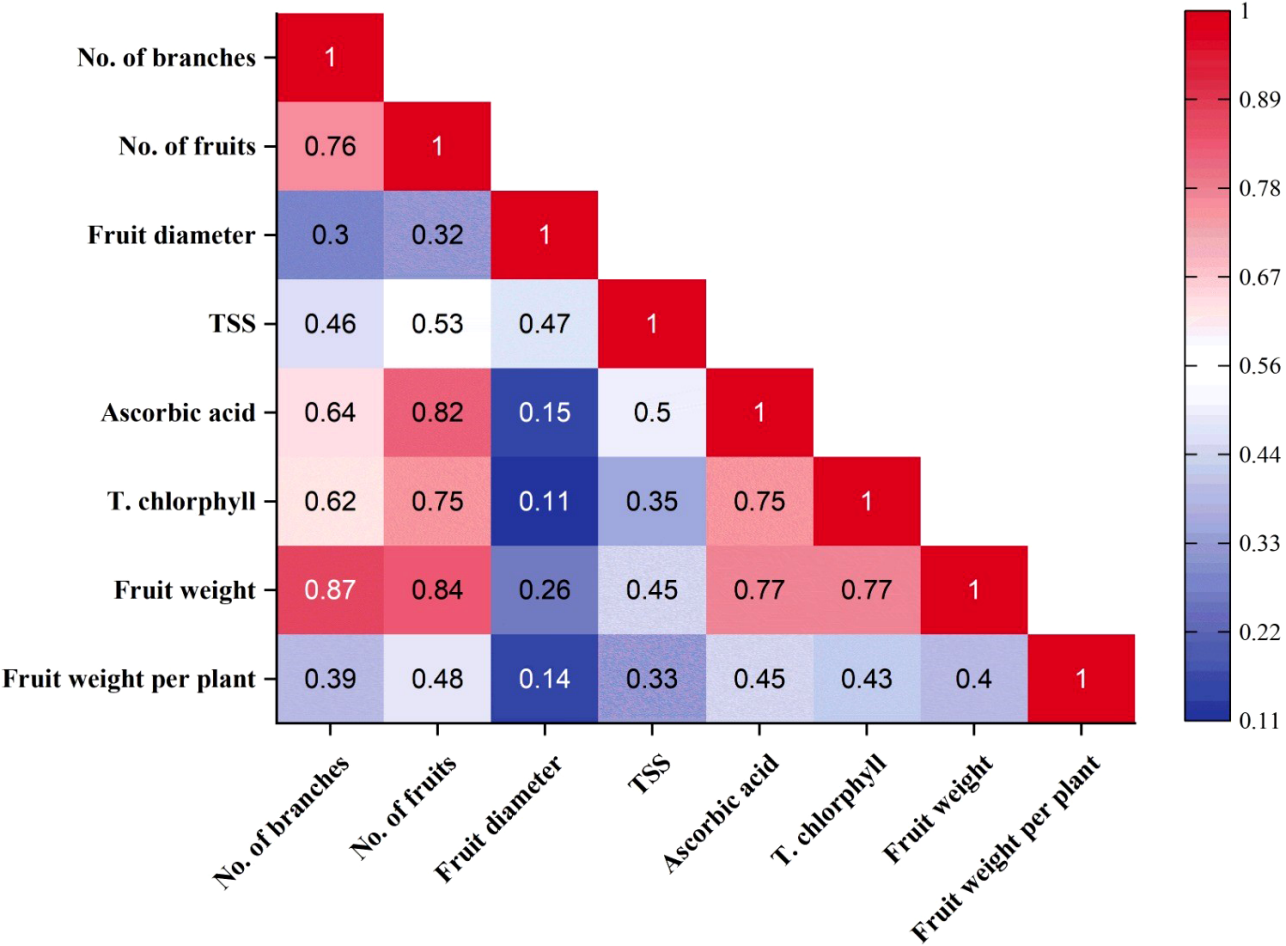
The relationship between the physical and chemical properties of sweet pepper plants.

To better understand the relationships among the measured variables, a correlation matrix was generated (**Figure 10**), revealing strong positive associations between chlorophyll content, fruit weight, fruit number, ascorbic acid, and TSS. This suggests that treatments that enhance one trait tend to improve others, reflecting systemic physiological improvement rather than isolated responses. These correlations support the hypothesis that nutrient efficiency and improved soil-plant interactions have a comprehensive impact on sweet pepper performance.

**Figure 11:**
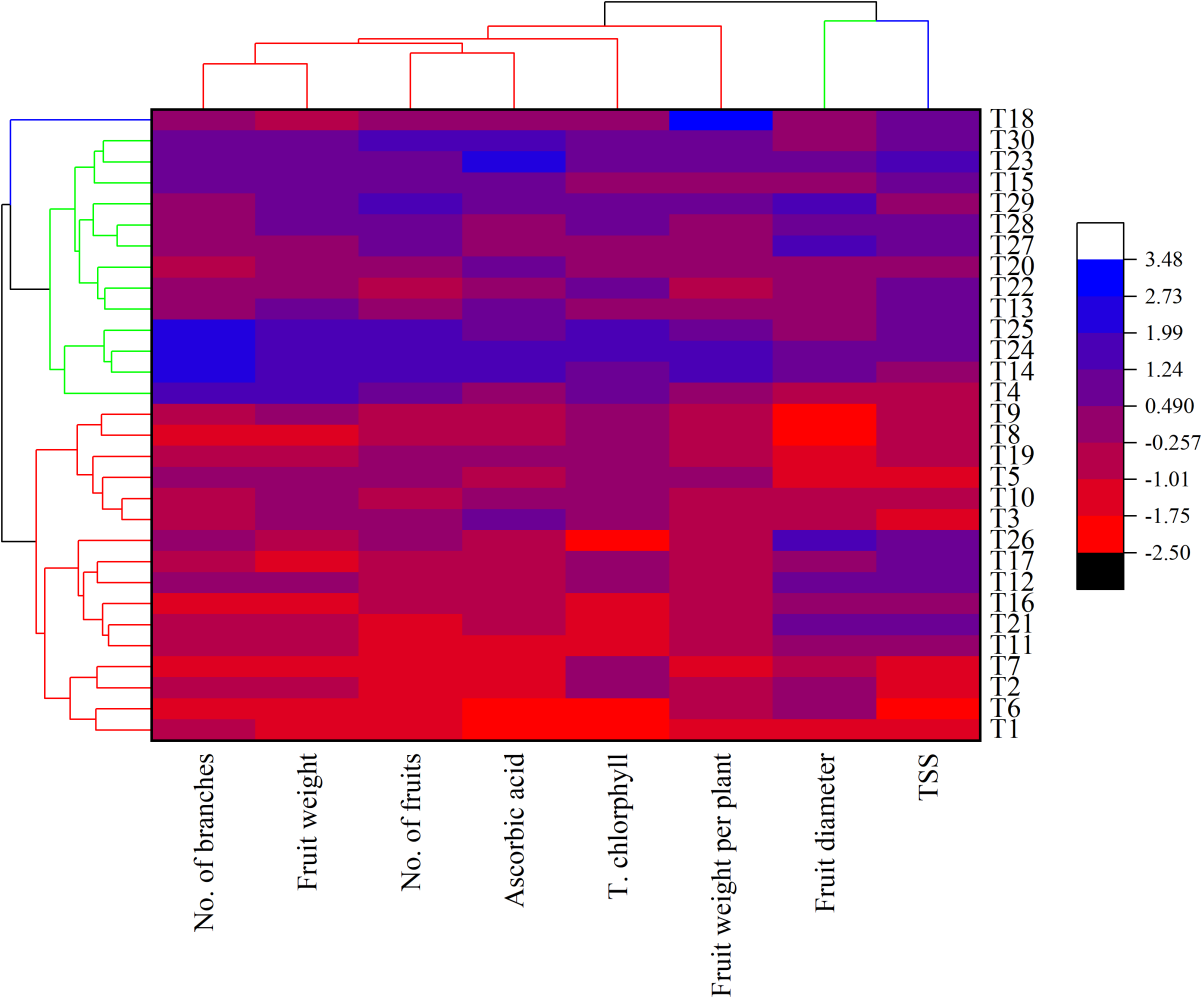
Heatmap correlation analysis of the chemical and physical attributes of sweet pepper plants treated with compost and nano-foliar spray. T1=Sand+ 0% Compost+ 0% Nano-foliar spray (NFS), T2=Sand+ 10% Compost+ 0% NFS,T3=Sand+ 15% Compost+ 0% NFS,T4=Sand+ 20% Compost+ 0% NFS,T5=Sand+ 25% Compost+ 0% NFS, T6=Clay+ 0% Compost+ 0% NFS, T7=Clay+ 10% Compost+ 0% NFS,T8=Clay+ 15% Compost+ 0% NFS,T9=Clay+ 20% Compost+ 0% NFS,T10=Clay+ 25% Compost+ 0% NFS,T11=Sand+ 0% Compost+ 1 cm^3^ L^-1^ NFS, T12=Sand+ 10% Compost+ 1 cm^3^ L^-1^ NFS,T13=Sand+ 15% Compost+ cm^3^ L^-1^ NFS,T14=Sand+ 20% Compost+ 1 cm^3^ L^-1^ NFS,T15=Sand+ 25% Compost+ cm^3^ L^-1^ NFS, T16=Clay+ 0% Compost+ 1 cm^3^ L^-1^ NFS, T17=Clay+ 10% Compost+ 1 cm^3^ L^-1^ NFS,T18=Clay+ 15% Compost+ 1 cm^3^ L^-1^ NFS,T19=Clay+ 20% Compost+ 1 cm^3^ L^-1^ NFS,T20=Clay+ 25% Compost+ 1 cm^3^ L^-1^ NFS, T21=Sand+ 0% Compost+ 2 cm^3^ L^-1^ NFS, T22=Sand+ 10% Compost+ 2 cm^3^ L^-1^ NFS,T23=Sand+ 15% Compost+ 2 cm^3^ L^-1^ NFS,T24=Sand+ 20% Compost+ 2 cm^3^ L^-1^ NFS,T25=Sand+ 25% Compost+ 2 cm^3^ L^-1^ NFS, T26=Clay+ 0% Compost+ 2 cm^3^ L^-1^ NFS, T27=Clay+ 10% Compost+ 2 cm^3^ L^-1^ NFS,T28=Clay+ 15% Compost+ 2 cm^3^ L^-1^ NFS,T29=Clay+ 20% Compost+ 2 cm^3^ L^-1^ NFS, and T30=Clay+ 25% Compost+ 2 cm^3^ L^-1^ NFS.

The heatmap cluster analysis (**Figure 11**) visually reinforces the superior performance of specific treatments. T24 (20% compost in sandy soil + 2 cm^3^ L^-1^ nano-fertilizer) emerged as the top-performing treatment across most traits, followed by T23 and T27. In contrast, control treatments (T1, T6), which received no compost or nano-fertilizer, performed poorly across all parameters. These visual insights provide strong evidence of the benefit of integrating organic and nano-nutrient strategies, especially in sandy soils where baseline fertility is low.

**Figure 12:**
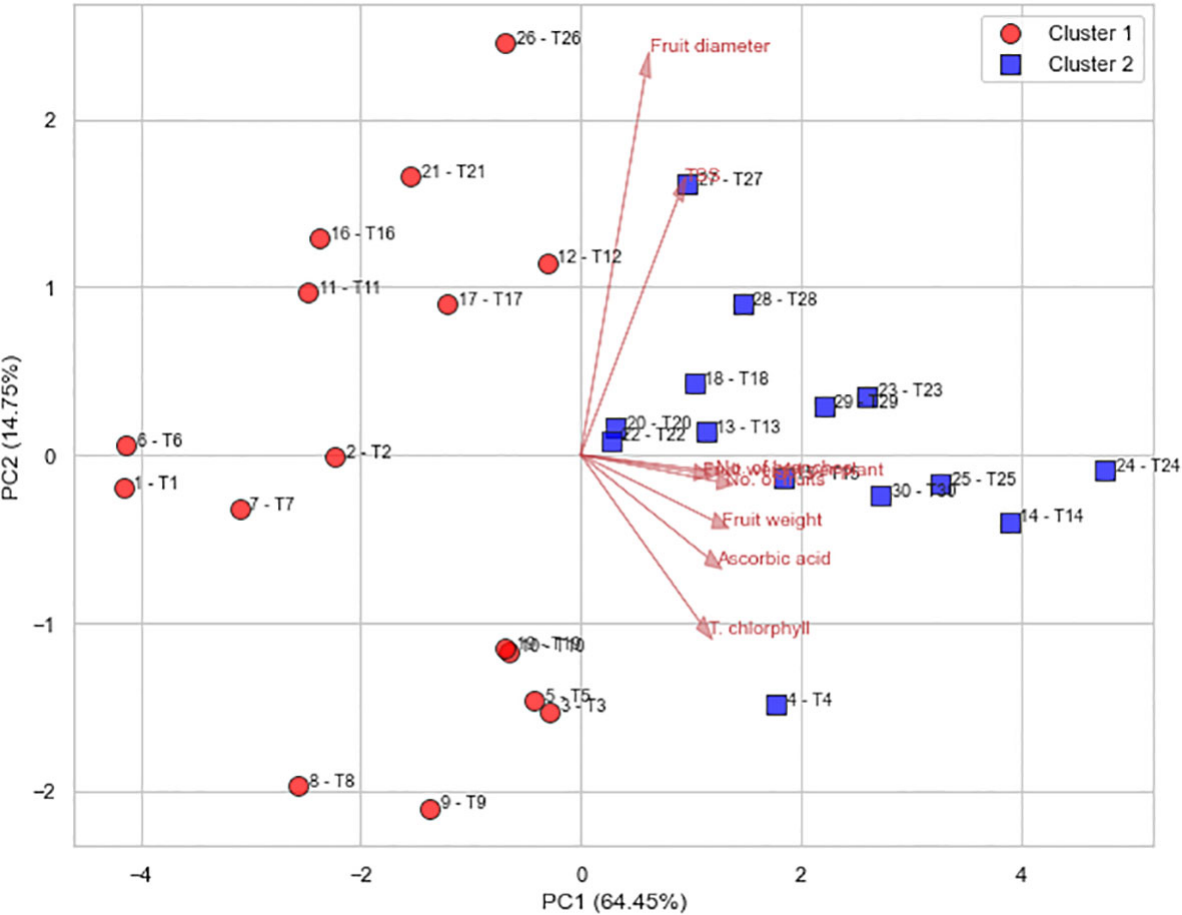
PCA biplot of treatments grouped by clustering.

Lastly, PCA (**Figure 12**) captured over 79% of total variability in the first two components, confirming the robustness of the dataset. Treatments were clearly divided into two distinct clusters, with high-performance treatments grouped in Cluster 2 (positive PC1), characterized by superior fruit weight, number, ascorbic acid, and chlorophyll content. T24 stood out as the most optimal treatment, highlighting its potential for adoption as a best-practice nutrient management strategy in greenhouse systems. Collectively, these results underscore the significance of integrating engineered compost and nano-fertilizer technologies for sustainable sweet pepper production. The findings confirm that compost produced via mechanical turning not only meets agronomic standards but also provides a sustainable and cost-effective organic amendment when combined with precision foliar nutrition. This approach enhances yield, fruit quality, and nutrient efficiency, offering a scalable and environmentally conscious solution for intensive greenhouse agriculture.

## 4 Discussion

### 4.1 Root-zone nutrient dynamics under compost amendment

Application of mechanically produced compost markedly improved sweet-pepper growth, chlorophyll content, and yield parameters. These findings agree with previous studies reporting that organic compost enhances soil physicochemical properties, increases cation-exchange capacity, and stimulates microbial activity responsible for nutrient mineralization. The engineered compost produced by the turning machine of Sayed et al. (2022) maintained an optimal C:N ratio and aeration level, accelerating microbial decomposition and releasing available N, P, and K to the rhizosphere. Enhanced microbial biomass and enzyme activity likely promoted ammonification and nitrification processes, improving N assimilation and root growth. Consequently, plants grown in compost-amended sandy soils exhibited higher chlorophyll and SPAD values, reflecting improved nitrogen uptake and chloroplast development (Arancon et al., 2003; Jamir et al., 2017; Azim et al., 2018). These results confirm that compost not only serves as a nutrient source but also acts as a biostimulant through its effect on microbial-driven nutrient cycling and root–microbe interactions.

### 4.2 Foliar nano-nutrient uptake and photosynthetic regulation

Foliar application of nano-fertilizer further enhanced physiological performance by improving nutrient penetration and translocation within leaf tissues. Nano-particles possess a high surface-to-volume ratio, allowing efficient diffusion through stomata and cuticular pathways. In this study, nano-Ca–Mg–B fertilization increased chlorophyll concentration, ascorbic-acid content, and total soluble solids, indicating improved photosynthetic efficiency and carbohydrate metabolism. Calcium plays a key role in stabilizing thylakoid membranes and activating photosystem II, whereas boron regulates carbohydrate transport and reproductive development. Hence, the observed increase in fruit quality and antioxidant levels can be attributed to enhanced ion homeostasis and redox balance within chloroplasts. Similar results were reported by Avila-Quezada et al. (2022) and Esringü et al. (2011), who demonstrated that nano-nutrient sprays elevated photosynthetic pigments and sugar accumulation through efficient foliar uptake.

### 4.3 Synergistic interaction between compost and nano-fertilizer

The integrated application of compost and nano-fertilizer produced the most pronounced improvements in physiological and biochemical attributes. This synergy arises from complementary nutrient pathways: compost enhances soil nutrient reservoirs and root uptake, while nano-fertilizer ensures rapid foliar nutrient supply and metabolic activation. The concurrent stimulation of root and shoot nutrition likely established a balanced C:N ratio and improved carbon fixation efficiency. Enhanced chlorophyll content under the integrated treatment signifies higher photosynthetic capacity, while elevated ascorbic acid and TSS reflect stronger antioxidant metabolism and sugar transport. These results align with Mukanov and Gulin (2024) and Channab et al. (2024), confirming that combining organic and nano inputs can synchronize macro- and micro-nutrient dynamics to achieve superior plant metabolic performance.

### 4.4 Influence of soil texture on physiological response

The response magnitude was greater in sandy than in clay soils, consistent with earlier findings that compost markedly improves water-holding capacity and nutrient retention in light-textured soils. In sandy soil, compost mitigated leaching losses and created a micro-environment favourable for microbial proliferation and root extension, enhancing nutrient accessibility and photosynthetic stability. Clay soils, in contrast, already possess high nutrient-holding capacity; thus, the relative improvement from compost–nano integration was less pronounced. This differential response highlights the importance of soil texture in regulating nutrient availability and physiological outcomes.

### 4.5 Overall physiological interpretation

The combined use of engineered compost and nano-fertilizer established a multi-level enhancement in nutrient uptake, photosynthetic metabolism, and antioxidant defence. Mechanistically, compost improved rhizosphere nutrient availability and microbial activity, while nano-nutrients intensified leaf metabolic activity. Their synergy fostered efficient nutrient partitioning between roots and shoots, reinforcing photosynthetic machinery and promoting metabolic homeostasis. This integrative mechanism explains the substantial improvement in both yield and fruit biochemical quality observed in greenhouse sweet pepper. Such physiological insights underline the potential of compost–nano integration as a sustainable strategy for optimizing nutrient efficiency in controlled-environment agriculture.

## 5 Conclusions

This study demonstrates the synergistic effect of mechanically produced compost (using a turning machine) and nano-fertilizer sprays on nutrient uptake, growth, and yield in sweet pepper (*Capsicum annuum L.*) under greenhouse conditions. These synergistic treatments improved physiological and metabolic responses at the leaf level and led to significant improvements in chlorophyll status, antioxidant activity, and yield, particularly in sandy soil conditions. Multivariate analyses, including principal component and correlation analyses, revealed strong associations between yield and key physiological traits related to photosynthetic efficiency and antioxidant capacity. These findings indicate that combining engineered organic amendments with nano-nutrient delivery can improve nutrient use efficiency and crop performance in protected cultivation systems. Overall, this approach provides a physiologically grounded and environmentally sustainable framework for greenhouse crop production, supporting resource-efficient agriculture and contributing to global sustainability objectives, including Sustainable Development Goals 13 (Climate Action) and 15 (Life on Land).

## Data Availability

The original contributions presented in the study are included in the article/supplementary material. Further inquiries can be directed to the corresponding author.
